# Retraction: Cefotaxime incorporated bimetallic silver-selenium nanoparticles: promising antimicrobial synergism, antibiofilm activity, and bacterial membrane leakage reaction mechanism

**DOI:** 10.1039/d4ra90146k

**Published:** 2024-12-11

**Authors:** Abdelrahman A. Elakraa, Salem S. Salem, Gharieb S. El-Sayyad, Mohamed S. Attia

**Affiliations:** a Botany and Microbiology Department, Faculty of Science, Al-Azhar University Nasr City Cairo 11884 Egypt salemsalahsalem@azhar.edu.eg; b Chemical Industries Department, Industrial Control Authority Cairo Egypt; c Department of Microbiology and Immunology, Faculty of Pharmacy, Galala University New Galala City Suez Egypt Gharieb.Elsayyad@gu.edu.eg Gharieb.S.Elsayyad@eaea.org.eg; d Drug Microbiology Lab, Drug Radiation Research Department, National Center for Radiation Research and Technology (NCRRT), Egyptian Atomic Energy Authority (EAEA) Cairo Egypt

## Abstract

Retraction of ‘Cefotaxime incorporated bimetallic silver-selenium nanoparticles: promising antimicrobial synergism, antibiofilm activity, and bacterial membrane leakage reaction mechanism’ by Abdelrahman A. Elakraa *et al.*, *RSC Adv.*, 2022, **12**, 26603–26619, https://doi.org/10.1039/D2RA04717A.

The Royal Society of Chemistry hereby wholly retracts this *RSC Advances* article due to concerns with the reliability of the data.

The EDX spectrum in Fig. 3f shows signs of potential manipulation. The spectra in Fig. 3e and f appear to overlay, with the addition and/or removal of some peaks in Fig. 3f. In Fig. 3f, one silver peak is presented at the incorrect energy level, whereas the lower shoulder peak is missing from the Se peak. The authors state that this was caused by contamination of the sample to be analysed by the EDX equipment, which could have been caused by interference with other samples.

The SEM/EDX mapping analysis for the Ag–Se NPs in Fig. 4 shows similarity to other SEM/EDX images published by the authors.^1^ Elements appear to have been added to the images published in this *RSC Advances* article, indicating signs of manipulation. The authors have not been able to provide a satisfactory explanation nor provide the raw images.

Given the significance of these concerns, the Editor has lost confidence that the findings presented in this paper are reliable.

This retraction supersedes the information provided in the expression of concern related to this article.

The authors were informed about the retraction of the article. Gharieb S. El-Sayyad has not agreed with the decision, the other authors have not responded.

Laura Fisher, Executive Editor, *RSC Advances*

2nd December 2024

**Reference**

1. A. I. El-Batal, M. Abd Elkodous, G. S. El-Sayyad, N. E. Al-Hazmi, M. Gobara and A. Baraka, *Int. J. Biol. Macromol.*, 2020, **165**, 169–186.

